# The Potential Use of Natural and Structural Analogues of Antimicrobial Peptides in the Fight against Neglected Tropical Diseases

**DOI:** 10.3390/molecules200815392

**Published:** 2015-08-24

**Authors:** Angélique Lewies, Johannes Frederik Wentzel, Garmi Jacobs, Lissinda Hester Du Plessis

**Affiliations:** Centre of Excellence for Pharmaceutical Sciences (PHARMACEN), North-West University, Potchefstroom 2520, South Africa; E-Mails: 20577966@nwu.ac.za (L.A.); 20134045@nwu.ac.za (W.J.F.); 22119027@nwu.ac.za (J.G.)

**Keywords:** antimicrobial peptides (AMPs), neglected tropical diseases (NTDs), malaria, parasites, antibacterial, antiviral, parasitic worms, innate immunity

## Abstract

Recently, research into the development of new antimicrobial agents has been driven by the increase in resistance to traditional antibiotics and Emerging Infectious Diseases. Antimicrobial peptides (AMPs) are promising candidates as alternatives to current antibiotics in the treatment and prevention of microbial infections. AMPs are produced by all known living species, displaying direct antimicrobial killing activity and playing an important role in innate immunity. To date, more than 2000 AMPs have been discovered and many of these exhibit broad-spectrum antibacterial, antiviral and anti-parasitic activity. Neglected tropical diseases (NTDs) are caused by a variety of pathogens and are particularly wide-spread in low-income and developing regions of the world. Alternative, cost effective treatments are desperately needed to effectively battle these medically diverse diseases. AMPs have been shown to be effective against a variety of NTDs, including African trypanosomes, leishmaniosis and Chagas disease, trachoma and leprosy. In this review, the potential of selected AMPs to successfully treat a variety of NTD infections will be critically evaluated.

## 1. Introduction

At the beginning of the new millennium, the international community, led by the United Nations, committed themselves to the Millennium Development Goals in order to drastically reduce extreme poverty and combat disease amongst the poorest populations of the world. Now, at the end of the 15 year period of this ambitious action plan, we can look back and evaluate the successes and shortcomings of this development framework. The sixth goal of this framework is to combat HIV/AIDS, malaria and other diseases. This goal has brought much-needed attention to neglected diseases, leading to the establishment of the Global Network for Neglected tropical diseases Control and a World Health Organisation (WHO) department, specifically tasked to address neglected tropical disease-related issues. Neglected Tropical Diseases (NTDs) are a diverse group of 17 disabling conditions, mostly affecting the world’s poorest populations [1]. These diseases affect more than 1.4 billion people around the world, causing more than half a million deaths annually [1,2]. Although these diseases distress a considerable portion of the global population, they may be considered “neglected” due to the dire lack of effective treatments and funding [3]. Despite all the positive progress from the Millennium Goals framework, NTDs still cause massive global suffering in millions of people. New treatment strategies are desperately needed to ease the burden caused by these tropical diseases.

The emerging and increasing resistance to antibiotics has become a threat to global public health and is driving novel research into the development of new antimicrobial agents. AMPs are promising candidates as alternatives to current antibiotics in the treatment and prevention of microbial infections [4,5,6]. Although there are a number of AMPs in clinical development [4,7,8], only a few have been successfully applied commercially. Perhaps the best known is the lantibiotic nisin (APD ID: AP00205) produced by the gram-positive bacterium *Lactococcus lactis*. This AMP exhibits antimicrobial activity against many gram-positive bacteria, including food-borne pathogens such as *Staphylococcus aureus* and *Listeria monocytogenes*, is not toxic to animals and was approved by the WHO in 1969 and the US Federal Food and Drug Administration (FDA) in 1988 for the use as a food preservative [9]. AMPs not only have broad-spectrum antibacterial activity, but also display antiviral and anti-parasitic activity [10] and therefore have potential for use in the treatment of a wide variety of NTDs. On the other hand, some of these microbes, for example the filarial worm *Onchocerca volvulus* [11], could prove to be a source of novel AMPs with therapeutic potential against microbial infections.

The aim of this review is to critically evaluate the potential of selected AMPs (both natural and structural analogues of natural AMPs) to successfully treat a variety of NTDs and malaria. NTDs that are covered include those caused by bacteria (leprosy/Hansen disease and trachoma), protozoa (chagas disease, human African trypanosomiasis and leishmaniosis), helminths (taeniasis and onchocerciasis) and viruses (dengue viral disease and rabies).

## 2. Antimicrobial Peptides

Antimicrobial peptides (AMPs) are considered natural antibiotics and are produced by all known living species, ranging from bacteria, fungi, and plants to invertebrates, non-mammalian vertebrates and mammals. To date, more than 2000 AMPs from various sources with a broad range of activities (ranging from antimicrobial activity to anticancer, spermicidal, chemotactic or antiviral activity) have been listed on APD2 [12], a database largely dedicated to natural AMPs [13,14]. For the scope of this review, only AMPs that are ribosomally synthesised [15] will be included in order to distinguish them from classical natural peptide antibiotics, such as vancomycin, which are assembled by non-ribosomal peptide synthetases [16]. Non-ribosomally synthesised AMPs can however be found in lower organisms such as bacteria, for example baceridin (APD ID: AP02372) which has recently been isolated from a plant-associated *Bacillus* strain [17,18]. Gene-encoded, ribosomally synthesised AMPs are evolutionary conserved components of the innate immune system, which serve as a first line of defence against microbial infections [19,20] and are therefore also referred to as host defence peptides. Foregoing the adaptive immune response (humoral and cell mediated immunity), the innate immune system can be mobilized against a variety of microorganisms, even if the host is encountering these microbes for the first time. After the initial infection, the interferon (IFN) response can be triggered by the recognition of foreign gene segments (this includes genomic dsRNA/DNA, mRNA and replication intermediates) or proteins by pattern recognition receptors (PRR). PRRs are divided into two families: (1) the cytoplasmic pathogen detectors which include the RNA sensitive retinoic acid-induced gene (RIG-I) and melanoma differentiation associated gene 5 (MDA5); and (2) the trans-membrane toll-like receptors (TLR) [21,22,23]. The recognition of alien genomic material by these PRRs triggers a complex cascade of cellular events which finally concludes with the deployment of immune regulatory proteins. Bacteria and fungi are also known to utilize cytolytic and antimicrobial peptides to obtain a competitive advantage over other micro-organisms in their habitat [24]. These peptides also play an important role in the innate immune system of most plants and animals [25].

### 2.1. Classification of AMPs

The selectivity and antimicrobial potency of AMPs towards microbes are determined by structural parameters including net charge and structural conformation [26,27]. Although structurally based classifications are generally used to divide AMPs into subgroups, AMPs may also be classified according to their net charge, which is determined by their amino acid composition.

#### 2.1.1. Classification of AMPs According to Net Charge

Based on the net charge, AMPs can be divided into anionic AMPs (AAMPs) and cationic AMPs (CAMPs). AAMPs are rich in aspartic and glutamic acid and consist of 5 to 70 amino acids with a net negative charge of −1 to −7 [28,29]. CAMPs are the most abundant form of AMP found in nature and are also the most thoroughly studied for therapeutic use. Hence, this review will primarily focus on the use of CAMPs for the treatment of selected NTDs. For a comprehensive overview of AAMPs, see Harris *et al.* [28]. CAMPs (from here on referred to simply as AMPs) typically consist of 12 to 100 amino acids with a net positive charge of +2 to +9 due to the excess of basic amino acids (arginine, lysine and/or histidine) compared to acidic amino acids [10].

#### 2.1.2. Classification According to Secondary Structure

**Figure 1:**
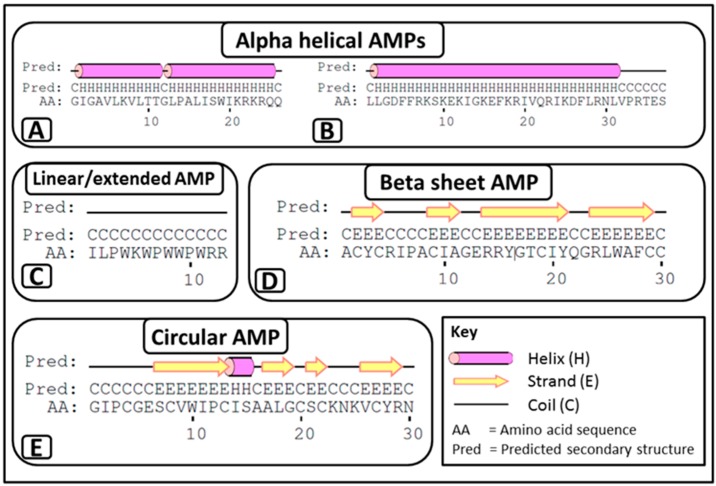
Secondary structures of the three major AMP classes. Examples of α-helical AMPs include (**A**) melittin, two α-helixes joined by a hinge between residues 11–12; and (**B**) LL-37, α-helix with an unstructured segment at the C-terminal; (**C**) indolicidin, which can be classified as an extended coil; and (**D**) human neutrophil peptide 1, which is an example of a β-sheet. In addition to the three major classes, circular AMPs including (**E**) circulin A, consisting of both α-helix and β-sheet structures, are also found in nature. Secondary structures were generated with the PSIPRED protein prediction server [33]. Disulphide bonds are not shown.

Despite being small in size, AMPs can be divided into three major structural classes (Figure 1) based on their secondary structure [27,30,31,32]:

i **α-helical AMPs**: These peptides are unstructured linear peptides free of cysteine residues that fold into α-helixes upon contact with membranes. They consist of approximately 50% hydrophobic residues, favouring an amphiphilic conformation upon interaction with membranes, which enables them to permeabilise microbial membranes. Some of these peptides are not strictly α-helical and may possess an internal kink and/or a flexible unstructured segment at the N- and/or C-terminus. For example, melittin (APD ID: 00146) (Figure 1A) from bee venom and the human cathelicidin LL-37 (APD ID: AP00310) (Figure 1B).
ii **Linear/extended AMPs**: These peptides are linear without cysteine residues and contain a high proportion of proline, arginine, glycine, tryptophan and histidine. Some of these AMPs form extended coils. Examples include indolicidin (APD ID: AP00150) (Figure 1C) from bovine leukocytes.
iii **β-sheet AMPs**: These peptides contain six to eight cysteine residues, forming two or more disulphide bonds, resulting in a stabilized β-sheet structure. For example α and β defensins such as human neutrophil peptide-1 (HNP-1, APD ID: AP00176) (Figure 1D) from mammals.

AMPs that do not typically fit into the previous three secondary structural classes are classified as circular AMPs [34]. The plant-derived circulin A (APD ID: AP00274) (Figure 1E) from the *Chassalia parviflora* species can be categorised into this group. Circulin A consists of a combined α-helix and β-sheet structure [35] and forms the so-called cyclic cysteine knot [36]. Though they display β-sheet-like structures, ϴ defensins can also be classified into this group, as they form cyclic octadecamers, which consist of two antiparallel β-sheets linked by three disulphide bonds [37].

The major forms of AMPs found in mammals are cathelicidins and defensins, although they are also found in non-mammals such as birds [38,39], fish [40,41], reptiles [42,43] and even plants [44] and insects [45]. Cathelicidins and defensins have been extensively reviewed elsewhere [46,47,48,49,50] and will therefore not be discussed in detail in this review.

### 2.2. Target Organisms and Mode of Action of AMPs

AMPs have broad-spectrum antibacterial, antiviral, antifungal and anti-parasitic activities. There are also AMPs that display anti-protist activity. A single AMP can have a single microbial target or have multiple microbial targets simultaneously, *i.e.*, displaying broad spectrum antibacterial activity, as well as being antifungal, anti-parasitic and antiviral (Table 1) [13,14].

**Table 1:** Antimicrobial peptides which display antibacterial (gram positive and negative) as well as antiviral, antifungal and anti-parasitic activity [13,14].

AMP	APD ID	Source	Sequence	Structure
Magainin 2	AP00144	African clawed frog *(Xenopus laevis)*	GIGKFLHSAKKFGKAFVGEIMNS	α-helix
Melittin	AP00146	Honey bee (*Apis mellifera)* (also *A. florea*, *A. cerana*)	GIGAVLKVLTTGLPALISWIKRKRQQ	α-helix
Dermaseptin-S1	AP00157	Sauvages leaf frog (*Phyllomedusa sauvagii*)	ALWKTMLKKLGTMALHAGKAALGAAADTISQGTQ	α-helix
HNP-1	AP00176	Human (*Homo sapiens*)	ACYCRIPACIAGERRYGTCIYQGRLWAFCC	β-sheet
LL-37	AP00310	Human (*Homo sapiens)* Chimpanzee (*Pan troglodytes*)	LLGDFFRKSKEKIGKEFKRIVQRIKDFLRNLVPRTES	α-helix
BMAP-27	AP00366	Domestic cattle (*Bos taurus*)	GRFKRFRKKFKKLFKKLSPVIPLLHLG	α-helix

#### 2.2.1. Perturbation of the Microbial Membranes by AMPs

**Figure 2:**
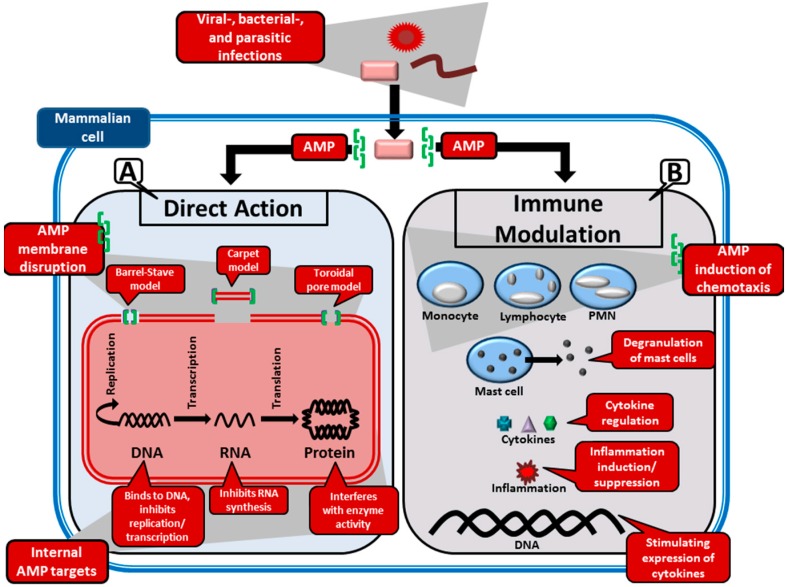
Main antimicrobial mechanisms of AMPs during the infection of a mammalian host cell. (**A**) The direct microbial action of AMPs involves the binding of these peptides to the microbial membrane/capsid, resulting in the infectious agent’s neutralization. There are three models proposed for this action: (i) the barrel-stave model, where the AMPs embed themselves into the membrane to form pores; (ii) the carpet model, which proposes that small portions of the membrane be removed by AMPs; and (iii) the toroidal model, which is similar to the barrel-stave model, but with the exception that the AMPs are permanently bound to the phospholipids in the membrane. Internal functions of AMPs may include DNA replication/translation inhibition, transcription inhibition and enzyme inhibition; (**B**) Many AMPs are also involved in immunoregulation. Some of these regulatory functions may include angiogenesis, cell proliferation, cytokine regulation, chemotaxis of certain leukocyte classes (including monocytes, lymphocytes and polymorphonuclear leukocytes or PMN), degranulation of mast cells, stimulation of phagocytosis and even modulation of gene expression.

Biophysical properties such as the secondary structure, net charge and hydrophobicity influence the interaction of AMPs with membranes as determined via experimentation with model and biological membranes [10,51,52]. Although, investigations of membrane mediated microbial activities of AMPs focuses mainly on bacteria [25,53], AMPs have also been found to display direct killing action against parasites [54], fungi [55] and enveloped viruses [56] through direct membrane or capsid interactions (Figure 2). The mechanism of interaction will differ depending on the microbial organism and the AMP under investigation, but generally the initial attraction between an AMP and the microbial membrane occur through electrostatic interactions. The cationic property of AMPs allow them to target negatively charged microbial membranes opposed to neutral zwitterionic phospholipid containing bilayer membranes of mammalian cells. The higher levels of cholesterol in mammalian cells can further be used as basis for AMPs to distinguish between microbial and mammalian cell membranes [53]. Although in most cases there seems to be no specific receptors for peptide binding, in the case of viruses, AMPs are thought to disrupt the viral capsid or interfere with host entry by binding to the viral glycoproteins [57]. Following binding of AMPs to cell membranes, cell death can occur by disrupting membrane integrity, resulting in the leakage of the cytoplasm, depolarization and osmotic imbalance, swelling of the cells and, finally, cell lysis [54,58,59].

Three main permeabilization models exist:

i **Barrel-stave model**: This model is used to describe the formation of pores in the lipid membrane. This is achieved through the amphipathic peptides that insert in a perpendicular orientation to the membrane and align so that the hydrophilic side chains point inward and form the transmembrane pore, while the hydrophobic side chains face outward and interact with the lipid bilayer [10,60]. This was the first model to be proposed as the mechanism of peptide induced pores [61]. AMPs that this model typically applies to are those that promote loss of electrochemical potential at low, well-defined peptide:phospholipid ratios [54]. The only AMP for which there is clear evidence of the barrel-stave mechanism is the non-ribosomally synthesised alamethicin (APD ID: AP02197), produced by the fungi *Trichoderma viride* [62].
ii **Toroidal model**: In this model, pores, consisting of peptides intercalated with lipids, are formed in the lipid membrane [63]. Amphipathic peptides accumulate parallel to the membrane surface, where their partial insertion causes the outer (but not the inner) leaflet to expand. The peptides re-orientate perpendicularly to the bilayer once a critical threshold is reached, thereby relieving tension. The bilayer undergoes a positive curvature and its thickness is modified, which creates transient pores consisting of both peptide and lipid molecules [54].
iii **Carpet model**: Partial membrane solubilisation in a detergent-like manner occurs with this model [54]. The amphipathic peptides accumulate parallel to the membrane via electrostatic interaction with anionic phospholipids—covering the membrane in a carpet-like fashion. When a threshold concentration is reached, detergent-like activity causes the formation of micelles and membrane pores [10].

It should be noted that although *in vitro* experiments have determined that some AMPs, for example magainin 2 (APD ID: AP00144) derived from the skin of the African clawed frog (*Xenopus laevis*), display cell selectivity towards microbial organisms without significant cytotoxicity towards mammalian cells [64,65], most AMPs should be considered as displaying “partial selectivity”. It is suggested that AMPs only display cell selectivity when placed in an actual situation where mammalian host cells are confronted with microbial organisms and that AMPs could potentially be toxic towards mammalian cells in the absence of microbial organisms. This fact is thought to be underestimated by current *in vitro* experimental procedures with regard to the volume of cells used for mammalian haemolysis (cytotoxic) *vs.* bacterial assays which does not give an accurate representation of the cytotoxic *vs.* the minimum inhibitory concentrations [26].

#### 2.2.2. Interaction of AMPs with Internal Targets

Although the main mechanism by which AMPs appear to directly kill microbial organisms is through membrane permeabilization, some AMPs do not damage microbial membranes but still manage to kill or inhibit the growth of these organisms. Alternatively, some AMPs may cause disruption of the microbial membrane without leading to inhibition or death of the microbial organisms [66,67]. AMPs are therefore also believed to display direct killing action through the interaction with anionic intracellular targets such as DNA and RNA (Figure 2) [25,68,69]. These AMPs still have to gain access to their internal targets through the cell membrane and are believed to translocate over the cell membrane of the microbial organism without causing significant damage to the membrane, although the precise mechanism is still not fully understood, as highlighted by Nicolas [68]. AMPs are thought to exert their intracellular activity through inhibition of nucleic acid and protein synthesis, inhibition of enzymatic activity, and the inhibition of cell wall synthesis and cytoplasmic membrane septum formation [25,69].

#### 2.2.3. Modulation of the Immune System by AMPs

Although the primary focus has been to identify AMPs with broad-spectrum antimicrobial activity through *in vitro* testing, many *in vivo* occurring AMPs have little direct antimicrobial activity [70]. The direct killing activities of some of these AMPs by either membrane perturbation or interaction with intracellular targets at physiological concentrations are antagonized by salt conditions, monovalent and divalent ions and serum [71,72]. Conversely, many of these peptides seem to be more involved in immunoregulation by acting as effectors of innate immune system. Some of these regulatory functions may include angiogenesis, cell proliferation, cytokine regulation, chemotaxis of certain leukocyte classes, degranulation of mast cells, stimulation of phagocytosis and even modulation of gene expression [70,73,74]. For example, human LL-37 has a pro-inflammatory action by up-regulation of specific chemokines (MCP-1 and IL-8) and binding to chemokine receptors such as IL-8RB, CCR, and CXCR-4 [75]. LL-37 receptors have been found in many different cell types, including monocytes, T-helper cells, mast cells, and epithelial cells, which suggest that certain AMPs can directly interact with host cells to induce immune responses [76,77]. However, it has also been shown that the induction of LL-37 has an *in vitro* anti-inflammatory function by inhibiting tumour necrosis factor α (TNF-α) production in macrophages [78]. These types of AMPs can counteract pro-inflammatory responses by preventing lipopolysaccharides (LPS) from inducing TNF-α production, thus suppressing the expression of LPS regulated genes in macrophages [75]. Apart from this indirect suppression, the LL-37 peptides can also induce specific anti-inflammatory genes by directly influencing macrophage gene expression, preventing an overwhelming immune response which could lead to fatal sepsis or endotoxemia [75]. Consequently, it seems that certain AMPs serve a dual, and seemingly counterproductive, immunoregulatory function by stimulating both pro-inflammatory and anti-inflammatory responses. This dualistic action may be a symmetric feedback mechanism to battle infection while simultaneously managing the septic levels by supressing the expression of certain cytokines. It is becoming clear that AMPs are a vital component of the germ line encoded innate immune system.

### 2.3. Therapeutic Potential and Obstacles Associated with AMPs

Many AMPs are promising candidates for drug development. The major advantages these peptides offer compared to conventional antibiotics are that they have a broader spectrum of antimicrobial action [10]; they exert their activity at micromolar concentrations [5,79] and they have a limited probability of inducing pathogen resistance [80]. Although some AMPs, such as melittin, have low therapeutic potential because of their characteristic cytolytic activity, these natural AMPs can be used as templates to create peptide analogues with increased therapeutic efficacy. Structural analogues of melittin have proven to display similar or higher levels of antimicrobial activity with reduced toxicity [81,82,83]. Hybrid peptide formulations could also improve the antimicrobial activity and reduce toxicity [84,85]. AMPs also play a role in modulating the immunity (both the innate and adaptive immunity in higher organisms) and using these naturally occurring AMPs as templates to design novel drug candidates for the selective up-regulation of the innate immunity and suppression of pro-inflammatory cytokine response could prove to be effective anti-infective therapies [86]. One of the major limitations for the use of AMPs is that they are peptide based, which makes systemic use difficult, due to bioavailability issues. Therefore, most AMPs in clinical development are being considered for topical application [4,8]. However, the use of nano- and microparticles has been shown to be beneficial for the oral delivery of insulin [87,88]. In cancer therapy, the use of nanoparticles, containing melittin, has a higher affinity towards diseased cells over healthy host cells [89]. A study by Piras *et al.* showed that the use of nanoparticles loaded with temporin B (APD ID: AP00095), an AMP from the European common frog, *Rana temporaria*, increased the antibacterial activity and reduced cytotoxicity against mammalian cells [90]. The use of AMPs encapsulated in nanoparticles should therefore be further investigated regarding its potential use in the treatment of antimicrobial infections. AMPs can also be considered for multi-drug treatment regimens, as synergistic interactions were observed when antibiotic and AMP combinations were used against methicillin-resistant *Staphylococcus aureus* (MRSA) [91]. Finally, AMPs have a broader range of activity, as conventional antibiotics cannot be used for the treatment of viruses and are less effective against most parasites, while several AMPs have been shown to display antiviral and anti-parasitic activities [37,54,57].

## 3. Neglected Tropical Diseases

According to the World Health Organization (WHO), Neglected tropical diseases (NTDs) are a diverse group of 17 disabling, though neglected, conditions mostly affecting the world’s poorest populations [1]. These diseases distress more than 1.4 billion people of low-income countries in Asia, Latin America and sub-Saharan Africa, causing more than 500,000 deaths annually [1,2]. In Africa alone, communicable diseases account for more than 70% of the total disease burden and deaths, with NTDs making up at least a fifth of this burden [92]. While these illnesses affect a considerable portion of the population, they may be considered “neglected” due to the dire lack of effective treatments and the fact that they are largely overlooked by funders and governing bodies (less than 5% of global health funding is allocated to alleviating the NTD burden) [3]. Although NTDs exist in the funding shadows of diseases like tuberculosis and HIV/AIDS, neglected diseases are noticeably contributing to the severity of the HIV pandemic by increasing the risk of horizontal and vertical HIV transmission [76,93,94]. While not all NTDs result in the loss of life, their morbidity and the accompanying economic loss are immense. NTDs can result in deformities and permanent damage, stunted growth, mental abnormalities and loss of eyesight and hence a lifetime of disability. The economic burden of NTDs are expressed as disability adjusted life years (DALYs), where each DALY unit refers to a healthy year lost due to premature death or disability [95]. The burden of NTDs amounts to staggering 46–57 million DALYs lost each year, second only to HIV/AIDS, with malaria and tuberculosis only coming in at third and fourth place, respectively [96].

The 17 NTD infections are caused by a wide variety of organisms including vector-borne protozoa (*Trypanosoma* and leishmaniosis), parasitic worms (helminths including *Taenia solium*, *Echinococcus granulosus*, *Echinococcus multilocularis*, *Wuchereria bancrofti*, *Opisthorchis* spp. *Fasciola* spp. and *Paragonimus* spp., *Onchocerca volvulus* and *Schistosoma*), bacteria (*Mycobacterium ulcerans*, *Mycobacterium leprae*, *Treponema pallidum*, *Treponema carateum* and *Chlamydia trachomatis*) and viruses (*Flavivirus* and *Lyssavirus*) [1]. Due to the initial lack of funding and the diverse nature of NTDs, research, diagnosis, prevention and treatment strategies are challenging. In late 2000, the international community committed itself to drastically reduce poverty and combat disease amongst poor populations of the world, known as the Millennium Development Goals. This action plan has brought much needed attention to the neglected diseases, leading to the establishment of the Global Network for Neglected Tropical Disease Control and a WHO department specifically tasked to address NTD related issues [3]. Despite all this positive progress, NTDs still cause massive global suffering in millions of people. Novel, cost-effective, rationally designed treatments are desperately needed to ease the burden of NTDs.

### 3.1. Neglected Bacterial Infections

The discovery of penicillin, the world’s first antibiotic, by Alexander Fleming in 1928 was a major milestone in the treatment of bacterial infections. However, an ever-increasing rise in antibiotic resistance amongst bacterial species has led research into the development of new therapeutic agents for bacterial infections [97]. AMPs are promising candidates in this regard [4,5,98]. As mentioned earlier, AMPs neutralize bacteria through direct killing actions which involve membrane disruption and interaction with intracellular targets [67]. NTDs caused by bacterial infections include leprosy and trachoma. Both these diseases have debilitating consequences that remain even after the disease is cured, contributing to the socio-economic burden of these diseases. Current treatment regimens involve the use of antibiotics, and although these prove to be effective the occurrence of antibiotic resistance could affect therapeutic outcomes in the future. This section will focus on the potential use of AMPs for the treatment of leprosy and trachoma.

#### 3.1.1. AMPs against Mycobacterium Infections and Hansen’s Disease

The mycobacterium genus includes pathogens that are known to cause serious infections in both humans and animals. Over 50 different mycobacterium species are known to be involved in human disease and most notably include Mycobacterium tuberculosis (causative agent of tuberculosis), *M. leprae* (leprosy) and *M. ulcerans* (Buruli ulcer) [6]. Mycobacteria are intracellular micro-organisms capable of infecting and replicating in host macrophages [99]. Due to the complexity and high lipid content of the mycobacterial membrane and emergence of multi-drug resistant strains, it is notoriously hard to effectively treat these infections [100]. Contributing to mycobacterium infections are the HIV/AIDS pandemic and the wide spread use of immuno-suppressive drugs [6]. Alternatives treatment strategies to conventional antibiotics may prove beneficial against this diverse genus of diseases.

Over the past two decades, numerous antimicrobial peptides, including LL-37, have been identified that offer direct and/or indirect antimicrobial activity against mycobacteria [101]. A large portion of the LL-37 peptide consists of hydrophobic amino acids, enabling this AMP to bind and disrupt the cell membrane of certain micro-organisms [46,102]. It has also been shown that during a mycobacterium infection, LL-37 is able to directly inhibit the host cell’s response by interacting with TLRs [103]. This AMP also plays a crucial role in the innate immune response to mycobacterium infections in several cell types [101]. In summary, the AMP, LL-37, has the capacity to fortify specific host innate immune responses, in addition to directly eliminating mycobacteria and/or inhibit their growth. AMPs like these may enable the development of novel and innovative antibiotics based on natural host antimicrobial peptides. Several AMPs play a central role in immune activation and inflammation regulation, possibly providing a unique foundation for the development of novel therapeutic agents.

“*And if the priest sees that the scab has indeed spread on the skin, then the priest shall pronounce him unclean. It is leprosy*.”

Leviticus 13:8

References to leprosy (Hansen disease)-like symptoms date back millennia and can be found in ancient Egyptian writings and those of Hippocrates [104]. This chronic human disease is caused by the bacteria *M. leprae* and can have potentially debilitating neurological effects [105]. Although the implementation of global multi-drug therapy programs has considerably reduced the burden of leprosy, areas like Africa, Latin America and India still report more than 200,000 cases annually [106]. Until recently, the origins of leprosy could only be speculated on, with written records found in India (600 BC), China (500 BC) and even the armies of Alexander the Great likely referencing this disease between 327–325 BC [107]. Today, through comparative genomic studies, it has been deduced that leprosy spread from Africa to India, Europe and the Americas, mainly along colonisation, migration and slave trade routes [104]. There are four distinct and regionally specific *M. leprae* strains found globally, with strain 1 mainly found in Asia, East Africa and the Pacific; strain 2 is less predominant and is only found in regions of Malawi, Ethiopia, Nepal and New Caledonia; strain 3 is found in America, Europe and North Africa; and strain 4 is mainly predominant in the Caribbean and West Africa [104]. *M. leprae* has a genome of 3,268,203 base pairs (bp) that encodes for 1604 proteins [108], a genome that is considerably smaller than that of *M. tuberculosis* [109].

Gerhard Henrik Armauer Hansen (1841–1912) dedicated his entire career to studying leprosy and identified *M. leprae* as the causative agent of this disease in 1873, which is also historically the first human-disease-causing bacterium to be identified [110]. Leprosy is a curable disease when treated with multi-drug therapy. Although it estimated that 95% of modern people have a natural immunity to leprosy, studies indicate that a small portion of the population is susceptible to this disease due to a defect in cell-mediated immunity [111]. In the 1940s, the sulfone drug, Promin (sodium glucosulfone) was introduced as the first effective treatment for leprosy and was replaced with dapsone (diaminodiphenyl sulfone) in the 1950s [112]. Nowadays, treatment consists of rifampicin and clofazimine once monthly while a dosage dapsone daily are used for treatment of patients with multi-bacillary infections over a period of 1 year [113]. Disadvantages of this modern treatment regimen include the long treatment periods and the looming prospect of antimicrobial resistance. Another major drawback, apart from understanding the complex clinical presentation of leprosy, is the lack of an appropriate experimental model.

AMPs appear to play an important role in the immune response to leprosy. The innate immune recognition of *M. leprae* seems to be mediated by the toll-like receptor 2 (TLR2) and mutations/polymorphisms in *TLR2* can affect host susceptibility to leprosy [114]. The cytokines interleukin (IL) 10 and 15 are also known to regulate macrophage functions in lesions [115]. The AMP, human β-defensin 3 (hBD3) (APD ID: AP00283), is also up-regulated during a leprosy type 1 reaction [106]. The induction of vitamin D dependant antimicrobial peptides LL-37, is one of the antimicrobial mechanisms employed by the host cell following phagocytosis [115]. In leprosy, LL-37 levels are relatively low due to the *M. leprae* mediated inhibition of the gene encoding for LL-37 (*CAMP*) [116]. This inhibition occurs through the up-regulation of the miRNA, has-mir-21, by *M. leprae*. Suppressing the translation of this miRNA, may lead to an improved innate immune response to *M. leprae*, enhancing the antimicrobial activity of LL-37. Another AMP involved in this disease is hepcidin (APD ID: AP00193). This defensin has a dual role as a hormone and antimicrobial peptide and plays a key part in hypoferremia of inflammation which increases the microbial resistance of host cells [117]. Hepcidin seems to be mainly up-regulated in multi-bacillary lesions, where it binds directly to the iron exporter efflux protein, ferroportin, resulting in its degradation [118]. By doing this, the host cell is limiting the circulating iron available to extracellular bacteria. Additionally, Stat3 is phosphorylated, which stimulates a downstream cytokine induced inflammatory response. Due to natural and artificial evolutionary pressures, pathogens, including *M. leprae*, are becoming antibiotic-resistant at alarming rates [119]. In order to combat the further emergence and spread of antibiotic resistance, new and innovative treatments strategies for microbial infections must be found. Pathogens are much less likely to successfully adapt their iron metabolic pathways to use alternative molecules after iron sequestration by AMPs.

#### 3.1.2. AMPs against Trachoma

Trachoma is one of the oldest recorded diseases of mankind, and although it has been eradicated from the developed world, it is still prevalent in poverty-stricken, underdeveloped countries. Areas where trachoma is endemic are usually overcrowded, lacking in proper public health care facilities, sanitation and access to clean water [120]. The disease is most prevalent in Asia, Africa, the Middle East, Australia and Central and South America [121]. Trachoma is the leading cause of infectious blindness globally and is also referred to as the leading preventable cause of blindness worldwide [122]. Currently it is estimated that in the 51 endemic countries, trachoma is responsible for the visual impairment of 1.8 million people, of whom 500,000 are irreversibly blind, and approximately 232 million people living in trachoma-endemic areas are at risk of infection [121].

This infection is caused by the gram-negative obligate intracellular bacterium *Chlamydia* *trachomatis*. It has no known animal reservoir and infection is transmitted to the eyes through physical contact with the discharge from the nose and eyes of infected people or flies (*Musca sorbens*), acting as passive vectors [122]. The life cycle of *C. trachomatis* is unique compared to other bacteria due to its parasite-like dependence on a eukaryotic host cell to complete its replication cycle [123]. During this bacteria’s life cycle, it adopts two distinct forms, the small extracellular elementary body (EB) and the larger intracellular reticulate body (RB). The EB is metabolically inactive and infectious, whereas the RB is metabolically active and represents the replicating form of *C. trachomatis.* The EB attaches to and enters host epithelial cells, transform into an RB, and exists inside inclusion bodies inside the host cell cytoplasm. The RBs use energy-rich metabolic intermediates from the host and undergo binary fission. Consequently, following the amplification of RBs, inclusions begin to take up a great part of the host cytoplasm and start to fuse. RBs differentiate back into EBs and are released through the lysis of host cells, infecting other cells [120,124]. The intracellular inclusions make it difficult for the immune system to eliminate the pathogen [120]. Two *C. trachomatis* biovars exist; trachoma to which serovars A-K are assigned and *Lymphogranuloma venereum* to which serovars L1, L2, L2a and L3 are assigned [125]. Serovars A, B, Ba and C in the trachoma biovar cause trachoma and serovars D-K are responsible for sexually transmitted genital tract infections [124]. Sexually transmitted *C. trachomatis* infections may be implicated in ocular trachoma infections in children [126].

The SAFE program for the treatment and management of trachoma has been established by the WHO [127], where the acronym SAFE represents: Surgery for trachomatous trichiasis, Antibiotics for active disease, Facial cleanliness to reduce transmission and Environmental improvements. Traditionally, antibiotic treatment consists of the use of 1% topical tetracycline ointment on both eyes twice daily for six weeks or a single oral dose of azithromycin. The treatment with azithromycin is favoured, as it has the potential to be used for mass community based treatment in endemic regions and displays higher patient compliance and effectiveness [128,129]. Much progress is being made in achieving the goal set by the WHO to eliminate blinding trachoma globally by 2020, with the disease being eradicated in some countries [121]. However, due to persistent infections and therefore antibiotic treatment, antibiotic resistance may become an issue. Therefore, alternative treatment strategies need to be investigated.

AMPs have been investigated for the use of the treatment of *C. trachomatis* infections. A study performed by Donati *et al*. showed that *in vitro* SMAP-29 (APD ID: AP00155), an α-helical cathelicidin from sheep (*Ovis aries*), was able to reduce the inclusion number for 10 strains of *C. trachomatis* (including serovars A, D, E, H and I). The integrity of EBs was also compromised [130]. Although, the direct therapeutic use of SMAP-29 is problematic because of its cytotoxic and hemolytic activity towards mammalian cells, this study indicates that SMAP-29 could be a useful compound in the development of anti-chlamydial drugs using this natural peptide as a template and making analogues with higher specificity and lower toxicity [131]. Another approach, which may be considered instead of the direct application of these peptides, is AMP gene therapy [132]. This approach uses recombinant plasmid vectors to express AMPs, which may potentially be cytotoxic to healthy host cells, in a controlled manner in infected cells. Studies performed by Lazarev *et al*. [133,134,135] demonstrated the potential use of AMP gene therapy for the treatment of *C. trachomatis-*infected cells. An *in vitro* study by Lazarev and colleagues showed that the introduction of recombinant plasmid vectors expressing the melittin gene, under the strict control of an inducible promoter, showed inhibition of *C. trachomatis* in infected cells. The mechanism by which the bacterial growth was inhibited was suggested to be through direct cytotoxic effect of melittin on the bacteria. It was also shown that the transmembrane potential of the transfected cells was lowered, which could disrupt the adhesion of the bacteria to the cell and hinder the normal process of intracellular development [133]. In a subsequent *in vivo* study by Lazarev *et al*., melittin was produced in the genital tract of mice infected with *C. trachomatis*, using the plasmid vector system. The majority of the melittin-protected mice were free of the pathogen after 27 days [135]. In a later *in vitro* study performed by the same group, the anti-chlamydial activity of the recombinant plasmid vector encoding cyto-insectotoxin 1a (CIT 1a, APD ID: AP02163), a linear cytolytic AMP found in the venom of the central Asian ant spider, *Lachesana tarabaevi*, was investigated [134]. This study concluded that CIT 1a was an attractive candidate for targeting intracellular pathogens, as *C. trachomatis* infection was inhibited in the early stages of its life cycle, with a higher efficacy than had previously been reported for melittin [133] and with a negligible effect on cell viability. The effect of the direct expression of melittin in infected cells on cell viability was, however, not reported for the previous two studies. Based on these results, CIT 1a can be considered a potential agent for gene therapy for *C. trachomatis* infections.

### 3.2. Neglected Protozoan Infections

All protozoa are unicellular eukaryotic organisms with a trophozoite stage and a resistant cyst form. Medically important phyla include apicomplexa, such as *Plasmodium* and *Toxoplasma*, sarcomastigophora such as *Trypanosoma* and ciliophora such as *Trichomonas*. The pathogenicity of protozoa is not well understood, but in general they have fewer pathogenic mechanisms than bacteria. They have the distinct advantage of being able to avoid host defences by several mechanisms. Transmission of protozoa to humans occurs through the faecal-oral route or through a vector [136,137]. Neglected diseases caused by protozoa include chagas disease, human African trypanosomiasis and leishmaniosis. Most of these protozoan diseases have enormous impact on human health and cause great economic and social burdens [138]. The typical protozoan infections are appealing targets for AMPs, as the molecules can be targeted to stages in the invertebrate vector or the vertebrate host. There are excellent reviews on the possibility of using AMPs as new chemotherapeutic options for malaria, chagas disease and sleeping sickness, as well as leishmaniosis [34,139]. This section will focus on the potential use of AMPs for the treatment of the neglected diseases chagas disease, sleeping sickness, leishmaniosis and malaria.

#### 3.2.1. AMPs against Chagas Disease

Chagas disease, or American trypanosomiasis, is caused by the parasite *Trypanosoma cruzi*. The disease was initially confined to South America, but 2010 epidemiological data include North-America, Europe, Australia and Japan as sites of *T. cruzi* infection [136,137,140]. The parasite is spread by the triatomine bug (part of the diverse Reduviidae family), which infects the human host when feeding, and it is also spread by travellers, organ transplants, blood transfusions, contaminated soil, food or water and by mother to infant transmission. The parasite has a complex life cycle including a trypomastigote in human blood and the epimastigote in the vector [140]. The clinical presentation occurs in two phases: an acute phase that lasts about two months after initial infection, and a chronic phase that can last for years. The acute infection is often misdiagnosed or unrecognized. However, treatment is most effective when initiated during the acute phase. From the intermediate phase, which is characterised by patients with chronic infection without clinical symptoms, 20%–30% of patients progress to a more serious chronic stage with chronic inflammation of the heart or digestive muscles [141]. There are only two drugs available for the treatment of chagas disease, benznidazole and nifurtimox. Both of these drugs are highly effective if administrated during the acute phase, but the efficacy declines with prolonged infection. Additionally, these drugs are toxic with negative side effects and are not effective in the chronic stage of infection [138,142,143]. There is a dire need for alternatives and AMPs from natural compounds seem to be an attractive option.

Several studies have reported promising effects of crude venom extracts containing AMPs from various sources against protozoan parasites. The crude venom of *Apes mellifera* was shown to decrease the viability and alter the ultrastructure of all *T. cruzi* developmental forms. This venom was also shown to be selective towards the parasite with no toxicity in mammalian cells at the tested concentrations [144]. In a follow up study by the same group, the AMP melittin from the venom of *A. mellifer*a was used to prove that the peptide induced morphological alterations in the different developmental forms of the parasite, which could be characterized as apoptosis and autophagy. They also provided evidence that melittin could be used in concentrations up to 1 µg/mL to treat infected host cells [145]. Another group investigated the efficacy of the antimicrobial peptides apidaecin 14 and melittin from *A. mellifera*, penaeidin (APD ID: AP00392) from the black tiger shrimp (*Penaeus monodon*), cecropin A (APD ID: AP00139) from the silk moth *Hyalophora cecropia*, moricin (APD ID: AP00147) from the silk worm *Bombyx mori* and magainin 2 produced by the African clawed frog (*Xenopus laevis*). Cecropin A, magainin 2, apidaecin and melittin were able to kill *T. cruzi*. These peptides were also investigated in combinations for synergistic effects, and in each case the addition of a second AMP increased toxicity [146]. Previously synthetic analogues of magainin 2, namely magainin B, G and H, were evaluated against *T. cruzi*. Only magainin B and G were effective against the parasite [147]. Synthetic derivatives of cecropin B were active against *T. cruzi*, where Shiva-1 the peptide with only 40% homology was most effective at killing promastigotes [148].

Dermaseptins and phylloseptins are cutaneous secretions of the tree frog *Phyllomedusa* genus. Dermasiptin-01 (APD ID: AP01389) from *Phyllomedusa oreades* showed significant lytic activity against *T. cruzi* with minimal activity against erythrocytes [149]. Dermaseptins form *Phyllomedusa nordestina* was investigated by another group. Dermaseptin 1 and 4 and phylloseptin-7 and 8 were effective against *T. cruzi* at micromolar concentrations. Dermaseptin 1 and 4 and phylloseptin 8 were also non-toxic to macrophages [150]. AMPs from various aquatic animals were also evaluated against *T. cruzi* promastigotes. Only tachyplesin I (APD ID: AP00214) from the horseshoe crab *Tachypleus tridentatus* was able to kill *T. cruzi* epi- and promastigotes at micromolar concentrations. Tachyplesin I was, however, hemolytic at high concentrations [151].

#### 3.2.2. AMP against Human African Trypanosomiasis

Human African trypanosomiasis is a parasitic disease mainly caused by two subspecies, *Trypanosoma brucei gambiense* and *T. brucei rhodesiense*, and causes chronic sleeping sickness in sub-Saharan African countries [136]. The clinical presentation of the disease is complex, making diagnostics challenging and treatment difficult [137]. The life cycle is similar to chagas disease with a trypomastigote phase in the human reservoir; however the epimastigote exists in the tsetse fly vector. Due to parenteral administration and severe toxicity associated with current chemotherapy options increasing incidence of treatment failure is observed [152]. The drugs used in the treatment depend on the infecting species as well as the stage of infection. Pentamidine and suramin is used in the first stage, and although effective, suramin has severe side effects. Second stage treatment includes IV melarsoprol, eflornithine and a combination of nifurtimox and eflornithine [153,154]. In comparison with the other parasitic infections, relatively few AMPs have been identified or evaluated for efficacy against *T. brucei*.

Temporin-SHd (APD ID: AP02118) from the North-African frog *Pelophylax saharicus* had significant growth inhibitory activity against *T. brucei* and *T. cruzi* [155]. Mammalian AMPs including α-defensins, β-defensins and cathelicidins have proved successful against *T. brucei*. *In vivo* administration of cathelicidins to mice infected with *T. brucei* significantly reduced parasitemia [156]. In another study the bovine cathelicidin BMAP-27 (APD ID: AP00366) from *Bos taurus* (bovine) and its derivative BMAP-18 inhibited both life cycles of *T. brucei* at low micromolar concentrations. BMAP-18 induced apoptosis-like cell death, but necrosis was induced at higher concentrations [157].

#### 3.2.3. AMPs against Leishmaniosis

Leishmaniosis species causes serious disease in humans. The typical infection is cutaneous, although visceral (kala-azar) and mucocutaneous leishmaniasis also exist. Visceral leishmaniasis caused by *Leishmania donovani* is fatal if left untreated. Cutaneous leishmaniasis caused by *L. major*, *L. tropica*, *L. mexicana* and *L. panamensis* frequently occurs every 3 to 18 months and leaves disfiguring scars [136,158]. Leishmania have a simple life cycle with only an amastigote in mammalian cells and promastigote in the insect (sand fly) [137]. The current chemotherapy available includes pentavalent antimonials sodium stibogluconate and meglumine antimoniate, amphotericin B and its lipid formulation AmBisome® (amphotericin B) and pentamidine. Antimonials remain effective for some forms of leishmaniasis, but the drugs’ usefulness is limited by the required parenteral administration for 28 days and the emergence of significant resistance. The use of pentamidine is limited due to its toxicity [159]. Several AMPs have been evaluated against *Leishmania* and some promising candidates have been identified.

Temporins, isolated from the European common frog, *Rana temporaria*, represent natural peptides with high anti-leishmanial activity. One of the first studies to prove the anti-leishmanial activity of temporins investigated temporin A (APD ID: AP00094) and B (APD ID: AP00095) against *L. donovani* promastigotes and *L. pifanoi* amastigotes. They found that both temporin A and B were active against the insect and mammalian stages at 15–25 µM concentrations [160]. Chadbourne *et al.* investigated the potential use of temporins against *L. mexicana* insect stage promastigotes and the mammalian stage amastigotes. The temporins investigated included temporins A, B and 1Sa. These AMPs showed no significant activity against *L. mexicana* amastigotes, but significant anti-leishmanial activity was observed against promastigotes with temporin A [161]. Temporin-1Sa (APD ID: AP00898) isolated from the North-African frog, *Pelophylax (Rana) saharica*, had significant activity against the promastigote and amastigote stages of *L. infantum* at a concentration that was not harmful to macrophages [162]. In another study temporin-Shd had similar activity against several species of Leishmania promastigotes and amastigotes [155]. Temporins A, B, 1Sa, F (APD ID: AP00098) and L (APD ID: AP00101) were effective against *L. mexicana* promastigotes, but the amastigotes were resistant to all temporins tested. The resistance was attributed to the lack of proteophosphoglycan and anionic charge in the membrane of the amastigotes [163]. The proposed mechanisms of action whereby they function include rapid disruption of plasma membrane potential and decrease in intracellular ATP levels [160].

Dermaseptin S4 (APD ID: AP00160) from the South-American Sauvages leaf frog (*Phyllomedusa sauvagii*) and its synthetic analogues induced potent lysis for *L. major* promastigotes. Positive or negative mono-substitutions in the synthetic peptides did not significantly affect the antileishmanial activity [51]. Dermaseptin 01 was shown to be active against *L. infantum* promastigotes by membrane damage and flagella alterations [164]. Dermaseptin from *Phyllomedusa hypochondrialis* (DShypo 01) was evaluated against *L. amazonensis*. It proved more effective at killing promastigotes than dermaseptin 01, and was not toxic to white blood cells or erythrocytes [165].

Other AMPs from various organisms active against *Leishmania* include gomesin (APD ID: AP00191) from the tarantula spider *Acanthoscurria gomesiana*, indolicidin and plant derived thionins. Gomesin decreased the viability of *L. amazonensis* promastigotes with little activity against human erythrocytes [166]. Indolicidin and two peptides derived from seminalplasmin exhibited significant anti-leishmanial activity. Additional to the membrane effects these peptides were also able to induce autophagy in *L. donovani* [167]. Different isoforms of thionins from common wheat (*Triticum aestivum*) were tested against *L. donovani* promastigotes. These thionins proved effective in the low micromolar range and membrane permeability was found to be an essential step in the lethal mechanism [168].

Also, synthetic cecropin A, andropin and dermaseptin were found to be active against *L. major* and *L. panamensis*. These three synthetic peptides have higher therapeutic potential were also highly selective with low toxicity against human erythrocytes and dendritic cells [169]. Pexiganan (Locilex^®^), a synthetic magainin-based lysine-rich peptide, which is undergoing phase 3 clinical trials for diabetic foot ulcers [4], induces apoptosis in Leishmania promastigotes. Activity is favourable if the strain of Leishmania is surface protease-deficient [170,171]. However, an arginine-rich variant of pexiganan proved to be protease resistant and displayed enhanced activity against wild type Leishmania *in vitro* [172]. These studies provide valuable insights into the use of AMPs against parasitic infections

#### 3.2.4. AMPs against Malaria

Malaria is an infectious disease caused by parasites of the *Plasmodium* genus. This life-threatening disease is responsible for 219 million new cases and 660,000 deaths annually, making it one of the deadliest modern infections [173]. These parasites are primarily hosted by female *Anopheles* mosquitoes, which act as vectors transmitting the protozoan organisms to humans when feeding. There are four known species that infect humans: *Plasmodium falciparum*, *P. vivax*, *P. ovale* and *P. malariae*, though *P. falciparum* causes the majority of malaria infections [136,173]. The parasite has a complex life cycle with an asexual cycle in the human host and a sexual cycle in the mosquito. The asexual cycle includes ring and trophozoite stages in red blood cells, which are targets for chemotherapy. The sexual stage in the mosquito includes gametocytes and sporozoites, which are transmitted to humans [137]. Clinically, the disease presents initially with flu-like symptoms, which may be difficult to recognise as malaria. If left untreated, within the first 24 h *P. falciparum* malaria may progress to a more severe illness. Chemotherapy, when initiated early, is successful, but increasing resistance to current treatment options is proving problematic [174]. Additionally, many antimalarial drugs are toxic in high doses, though Pheroid vesicles have been shown to reduce toxicity [175]. Various AMPs have been investigated as a possible new class of antimicrobial drugs. A very thorough review on this topic was recently published [34], though only selected examples will be highlighted in this section.

The hemolytic dermaseptin S4 was shown to have antimalarial activity against *P. falciparum* (FCR3 strain). The peptide causes significant lysis of the ring and trophozoite stages of the parasite [176]. Natural peptides melittin and transportan 10 (TP10), mastoparan X (APD ID: AP02355) and anoplin (APD ID: AP00447) from the *Vespula lewisii* wasp, were effective against *Plasmodium* sporogenic stages and were considered to block transmission [177]. Synthetic analogues of cecropin B, SB-37 and shiva-1 were effective at limiting the growth of different stages of *P. falciparum* [148].

Gomesin was evaluated against *P. falciparum* and *P. berghei*. This AMP inhibited chloroquine-sensitive (3D7) and chloroquine-resistant (W2) parasites, but only at micromolar levels, compared to nanomolar levels of artesunate. Gomesin was more effective against *P. berghei* mature gametocytes and also proved successful as a transmission blocking agent [178]. These examples of AMPs all act against different life cycles of the parasite, with different mechanisms of action. At this stage the most promising candidates in AMPs for malaria seems to be those that are involved in transmission-blocking. However, this is still a developing field and many of the AMPs active against other parasites have not been evaluated in malaria models.

### 3.3. Neglected Helminth-Related Infections

Helminths, also known as parasitic worms [179], are a group of evolutionary unrelated organisms that can be divided into two major groups, namely the Platyhelminthes (flatworms) and Nematoda (roundworms) [180,181]. Worldwide, more than 2 billion people are infected with one or more of these parasitic worms, including the hookworm, *Necator americanus*; the roundworm, *Ascaris lumbricoides*; and *Ancylostoma duodenale*; and the whipworm, *Trichuris trichiura* [182]. Although these parasitic infections primarily affect underdeveloped populations with insufficient sanitation, housing, water supplies and primary health care systems [183,184], they are also widespread in developed countries [185].

Presently, the treatments for helminth infection are parasite-specific and involve regular administration of anti-parasitic drugs. Although these treatments (including benzimidazoles for hookworm infections and praziquantel for schistosomiasis infections) are currently effective, parasite re-infections are still a problem and helminths will most likely develop resistance to these drugs [186]. Consequently, investigations into novel helminth-specific treatments should seriously be considered. There are currently eight helminth infections identified by the WHO as NTDs. This section will focus on the potential use of AMPs for the treatment of taeniasis, cysticercosis and onchocerciasis.

#### 3.3.1. Possible AMPs against Taeniasis and Cysticercosis

Taeniasis is a parasitic intestinal infection caused by two species of adult tapeworms, namely *Taenia solium* (pork tapeworm) and *Taenia saginata* (beef tapeworm). This parasitic disease is contracted when humans ingest raw or underprepared beef (infected with *T. saginata*) or pork (*T. solium*). The accidental consumption of *T. solium* eggs, through contaminated food or water, can lead to cysticercosis [187]. The eggs move into the digestive tract, where they hatch and develop into larvae (cysticerci). These larvae can then enter the circulation and invade host tissue and organs, such as muscles, eyes, skin and the central nervous system. If the cysticerci spread to the brain, neurocysticercosis may develop, which can be a fatal condition [188]. Symptoms include blindness, headache, dementia, meningitis and epilepsy. Current treatment of taeniasis includes praziquantel or niclosamide [189]. Treatment of neurocysticercosis includes long courses with praziquantel or albendazole, and also supporting therapy with anti-epileptic drugs and corticosteroids [190]. There are, however, drawbacks involved when using these drugs. Praziquantel can induce epileptic seizures in patients with neurocysticercosis, whereas niclosamide is only effective against adult intestinal tapeworms [187,191].

Temporin A and iseganan IB-367 (a protegrin-1 (APD ID: AP00195) derivative that belongs to the cathelicidins family) have anti-parasitic effects against *T. crassiceps* [192], a parasite which has a close relationship with *T. solium.* Iseganan IB-367 functions by disrupting cell membranes through the induction of pores. Both these peptides reduce the parasitic load and damage the tegumentary surface of the cysticerci. In a study by Landa *et al.*, temporin A reduced the parasitic load by 50% and iseganan IB-367 by about 25%. These peptides also induced morphological changes *in vitro* in the cysticerci [193]. AMPs have therefore been shown to be effective in destroying cysticerci and damaging the wall of the cysticerci, which is critical for the death of the cestode. These, or structurally similar peptides, should be investigated as alternatives to current anthelminthic treatment to bolster the treatment of taeniasis and cysticercosis.

#### 3.3.2. AMPs from Onchocerciasis

Onchocerciasis, also known as “river blindness”, is caused by the filarial worm *Onchocerca volvulus* [194], the second most common cause of blindness from infection after trachoma [195]. The disease is transmitted by the bites of infected blackflies (*Simulium* species) which breed in fast flowing streams and rivers, predominantly in central Africa [196]. The adult worms produce microfilariae in the human body where it migrates to the eyes, skin and other organs. Symptoms develop as a result of the microfilariae moving around in the body and inducing inflammatory responses. Severe itching, skin lesions and nodules under the skin are some of the symptoms that infected people develop. Eye lesions can also develop, causing visual impairment ultimately leading to permanent blindness. The recommended treatment for this disease includes ivermectin once yearly for 10 to 15 years [194]. Repeated doses of ivermectin over several years are required for the elimination of an onchocerciasis infection to halt the transmission of the parasite in the long term [197].

Human neutrophil peptide 1–3 (HNP1–3) was identified in *O. volvulus* worms extracts [198]. Human neutrophil peptides are α-defensins that belong to the family of cationic trisulfide-comprising antimicrobial peptides [199]. HNP1-3 has been shown to mediate the macrophage response to micro-organisms by stimulating the release of IFN-γ and TNF-α [200]. Defensins can neutralize a target micro-organism by binding and permeabilizing its membrane. Although human neutrophil peptides can still bind to the surface of *O. volvulus*, this parasitic worm may have developed a limited resistance to these AMPs through co-evolution. This may explain why human neutrophil peptides were isolated with *O. volvulus* worm extracts, as the peptides are able to bind to the surface of the worm but not cause permeabilization and death. Identifying and/or investigating natural AMP homologs of human neutrophil peptides may yield alternative treatments for onchocerciasis.

In another study, Eberle *et al.* identified at least three excretory/secretory products (including galectin) of *O. ochengi* and *O. volvulus* with significant antibacterial activity against *E. coli* [11]. The excretory/secretory peptides of many parasites are similar to host defence peptides and, as mentioned earlier, host defence peptides have been shown to have antibacterial, antifungal, anti-parasitic and antiviral activities [201]. The peptides excreted/secreted by *O. ochengi* and *O. volvulus* present a promising pool of potential novel AMPs and further investigation into these peptides is needed [11].

### 3.4. Antiviral Peptides and Their Potential Use to Treat Viral Diseases and Possible Application to Viral Neglected Tropical Diseases

Viral diseases are one of the leading causes of morbidity and mortality globally, especially in lower income countries. Adding to this problem, antiviral treatments are expensive to develop and are usually only effective against a single virus [202]. The control of viral diseases has always been a challenge, due to their genetic diversity, short and effective replication cycles, diverse transmission means, and adaptability, as well as the wide variety of hosts. In their history, the US Food and Drug Administration (FDA) has only approved about 60 antiviral drugs for seven viruses (cytomegalovirus, herpes simplex virus, hepatitis B and C viruses, human immunodeficiency virus-1 (HIV-1), influenza, and varicella-zoster virus) and almost half of the approved antiviral drugs are for HIV-1 [203]. Despite this, a considerable amount of peptides have been shown to be effective against wide range of viral infections [204,205,206,207,208]. There seem to be two main antiviral mechanisms of AMPs as reviewed in Klotman and Chang [57]. The first mechanism is similar to the antibacterial activity of AMPs and involves direct disruption of viral envelopes or interaction with internal viral targets, while the second is thought to be an indirect antiviral action by stimulating specific innate immune mechanisms of the infected host cell.

When the first antiviral AMP (HNP-1) was discovered in 1986, it was believed that these peptides only had a direct effect on enveloped viruses while overlooking non-enveloped viruses [209]. HNP-1 was reported to have a direct inhibitory effect on the enveloped viruses such as herpes simplex virus-1 and 2, influenza virus and vesicular stomatitis [209]. It is hypothesised that the method of direct inactivation is the disruption of the viral capsid or preventing host entry by binding to the viral glycoproteins [57], but the exact mechanism is not clear and requires further investigation. AMPs (including HNP-3 and human β-defensin-3) have no detectable direct effect on the non-enveloped rhino-, echo- and reoviruses [209,210]. However, it has been shown that the AMPs HNP-1 and β-defensin-3 can stimulate infected host cells to subdue the viral replication of non-enveloped viruses after virion entry [211]. Instead of blocking the binding of the virus to the host cell and subsequent endocytosis, antiviral peptides may obstruct the release of the virion from the endosomes. Still, further studies are needed to elucidate the mechanisms by which AMPs can suppress non-enveloped viral replication.

In a study done by Carriel-Gomes *et al.*, they evaluated the *in vitro* antiviral activity of nine AMPs against the human adenovirus (respiratory strain), type 1 herpes simplex virus and rotavirus (SA11 strain) [212]. Although several AMPs tested showed promising antiviral activity, most peptides were cytotoxic at their active concentrations. However, modifying the peptide structures of these AMPs may reduce their cytotoxicity and make them attractive antiviral treatments. In order for the cell to mount an effective antiviral offensive, most viral infections lead to the stimulation of a complex cascade of host cell signalling pathways (including Jak-STAT- and Toll pathways), ultimately resulting in the expression of IFN-stimulated genes (ISGs), which is able to directly inhibit viral replication [213,214,215]. The secretion of cytokines belonging to the interferon (IFN) family (IFN type I and III in particular), play an important role in this innate immune response by activating the expression of IFN-stimulated genes (ISGs) [216,217]. Many AMPs have been shown to exhibit antiviral activity during several phases of viral pathogenesis [218] and several AMPs play a central role in immune activation and inflammation regulation [77,219], possibly providing a unique foundation for the development of novel therapeutic agents. This section will focus on the potential use of AMPs for the treatment of dengue viral disease and rabies.

#### 3.4.1. AMPs against Dengue Viral Disease

Dengue viral disease is a mosquito (*Aedes aegypti*)-borne pathogen predominant in tropical and sub-tropical regions, putting more than 3.6 billion humans at risk of infection [220,221]. This disease is caused by one or more of five serotypes of the dengue virus (DEN-1 to -5) belonging to the genus *Flavivirus* [222,223]. Genetically, at least four of the five dengue virus serotypes seem to share a common ancestor in sub-human primates from central and east Africa [224]. The modern dengue virus is an enveloped, single positive-stranded RNA virus with a genome of ~11,000 bp that codes for three structural proteins and seven non-structural proteins [225]. Phylogenetic analysis indicated that dengue viruses have exceptionally high mutation rates (as high as 1 nucleotide mutation per life cycle), which is most likely due to the lack of proofreading ability in their RNA-dependant RNA-polymerase [226,227]. Only the female mosquitos act as vectors for the dengue virus and they are infectious throughout their entire lifespan [228]. Alarmingly, there has been a rapid ecological expansion of the virus’s vector, *A. aegypti*, over the past few decades, dramatically increasing the risk of human infection [221]. Dengue virus infects approximately 500 million people across 124 countries annually. The resulting infection causes nearly 21,000 deaths and an economic burden of more than US $950 million each year [220,229,230]. The majority of mortalities are caused when dengue viral disease progresses to severe dengue haemorrhagic fever (DHF) or dengue shock syndrome (DSS) [220]. Due to the five diverse dengue virus serotypes, high mutation rate and lack of a working animal model, no effective vaccine exists for this virus [231]. Additionally, disease prevention is impractical due to difficult vector control, and clinical treatment is limited to secondary shock prevention care. There is a dire need for the development of alternative anti-dengue viral treatments.

As an alternative approach, Rothan and colleagues investigated the potential of the AMP latarcin (Ltc-1; APD ID: AP01010) from the venom of the ant spider *Lachesana tarabaevi* to inhibit the replication of dengue viruses in cultured Vero cells [232,233]. In dengue virus, Rothan *et al.* showed that the Ltc-1 peptide binds to the non-structural protein 3 (NS3) which may inhibit substrate binding to the NS3 active site, hindering the formation of the NS2B co-factor active site [233]. For the release of the mature dengue viral structural and non-structural proteins, the NS2B-NS3pro complex must first cleave the dengue viral poly-protein at specific sites [234,235]. Consequently, the Ltc-1 inhibition of the NS2B-NS3pro complex can halt virus replication by hindering the post-translational function of the dengue viral poly-protein [233,236].

In another study, two synthetic AMPs designed to target the domain III of the dengue DENV-2 E protein significantly inhibited virus entry in to LLC-MK2 cultured cells [237]. Efficient entry of the virus particle into the host cell is crucial to the success of any infection. In the case of the dengue virus, attachment to the host cell surface receptors and subsequent viral–cell membrane fusion is mediated by the virus’s E protein [238]. The E protein consists of three domains comprising domain I (the central structural domain), domain II (the dimerization domain) and domain III (the receptor-binding domain) [239]. Apart from cell entry, the E protein is also the chief target for protective antibodies against the dengue virus, and there are several conserved regions between the dengue serotypes [238]. The two synthetic AMPs designed by Alhoot and colleagues targeted a short amino acid sequence in the lateral loop of domain III in strain 2 of the dengue virus. This may reduce the viral load during early infection and buy time for an effective immune response which can reduce the severity of infection. This inhibitory function of these synthetic AMPs against dengue virus can provide a basis for identifying similar natural AMPs to develop new therapeutic strategies against dengue infections.

#### 3.4.2. AMPs against Rabies

Rabies has an exceptionally high fatality rate for an infectious disease (fatality rate of virtually 100% after symptoms appear) and is responsible for an estimated 55,000 deaths annually [240]. However, due to underreporting, deaths may be as much as 100 times higher [241]. This zoonotic disease has been plaguing mankind for more than 5000 years and has a very unique and wide range of hosts for a viral disease, as it can infect almost all warm-bodied animals [242,243]. Rabies mainly affects the poor, rural communities of developing regions and is 100% vaccine preventable. The causative agent of rabies is the enveloped, negative-sensed, single-stranded RNA Lyssavirus (Lyssa, Greek goddess of madness and frenzy) from the *Rhabdoviridae* family [244]. The Lyssavirus genome consists of approximately 12,000 bp and encodes for 5 viral proteins. The virus is found in the saliva of infected animals and is usually transmitted through their bites. The rabies virus can supress the host’s innate immune response by subduing the type I interferon response [245]. After the initial wound entry, Lyssavirus uses the central nervous system to invade the host’s neurons, where it replicates and spreads [246]. Symptoms of the classical rabies infection commonly include hydrophobia, muscle spasms, extreme aggression (erratically attacking objects or other humans) and terror [242,247]. Nowadays, rabies infections are primarily controlled by limiting animal infections. In the United States, if a human is exposed to a rabies-infected animal, a prophylaxis containing rabies immunoglobulin together with an inactivated rabies virus vaccine is administered [248]. With the approval of the WHO, viral vector vaccines and DNA vaccines have been introduced in developing countries to reduce vaccination costs [249,250]. Pre-exposure rabies vaccines exist, but are not widely used and mainly reserved for individuals that have a high risk of infection, including researchers and veterinarians due to their high costs [251].

In a study by Real *et al.*, short, natural peptides that target rabies viruses were evaluated for their antiviral potential [252]. They proposed an antiviral drug discovery strategy based on the mimicry of natural peptides such as the lebocin 1 and 2 (APD ID: AP00359) from silkworms (*Bombyx mori*) and µ-conotoxin from cone snails (*Conus geographus*). A large amount of peptides were screened for their binding affinity to the phosphoprotein of the rabies virus. The viral phosphoprotein plays an important role in the transcription-replication complex and reduces non-specific RNA binding by acting as a chaperone for the nucleoprotein [253,254]. Only selected peptides with high binding affinity were then evaluated for their antiviral potential against rabies viruses. After an *ex vivo* inhibition of viral replication assay, they identified four structurally diverse peptides (C2, C6, C8 and P16) that exhibited strong rabies virus inhibitory properties [252,255]. Three of the four AMPs (C6, C8 and P16) had an algorithmically predicted α-helical conformation, while the AMP with highest antiviral activity, C2, seemed not to possess a helical-like structure. The central role that the viral phosphoprotein plays in the transcription-replication complex makes this protein an attractive target for antiviral treatments. Real *et al.* showed that AMPs based on natural peptides can be used to drastically inhibit the replication of rabies viruses. Their work also offers an alternative strategy for identifying novel antiviral peptides, with the objective to develop new types of antiviral treatments. Besides this study, very little published work is available on the antiviral treatments for rabies. There have, however, been some AMPs identified for other viruses in the *Rhabdovirus* family, including casein and α_s2_-casein, which can drastically inhibit the replication of the infectious haematopoietic necrosis virus in salmonid fish [256]. Another study also found that human α-defensin-1 has antiviral activity against the haemorrhagic septicaemia virus [257]. All these findings suggest that AMPs are a viable antiviral treatment option for viruses in the *Rhabdovirus* family, and more efforts should be made to identify and investigate these peptides as possible rabies treatments.

## 4. Conclusions

NTDs are a diverse group of 17 disabling, though neglected, conditions affecting a fifth of the world’s population, resulting in more than 500,000 deaths annually. These infections, prioritized by the WHO, are caused by a wide variety of organisms including vector-borne protozoa, parasitic worms, bacteria and viruses. Due to the diverse nature of NTDs and the initial lack of funding, research, diagnosis, prevention and treatment strategies are challenging. The Millennium Development Framework is a United Nations-led coalition for the alleviation of poverty in the developing regions of the world by 2015. One of the goals of this framework is to combat HIV/AIDS, malaria and Neglected tropical diseases. Although this goal has brought much-needed attention to neglected diseases, NTDs still cause massive global suffering in millions of people. Innovative, cost-effective, rationally designed treatments are desperately needed to ease the burden of NTDs.

Due to the ever-increasing rise in antibiotic resistance, there is a dire need for the development of alternative treatment strategies. AMPs, produced by all known living species, may be considered natural antibiotics due to their central role in the innate immune system, providing the first line of defence against microbial infections. These peptides have a broad range of activity against bacteria, parasites and viruses. Numerous studies have focused on the potential use of AMPs for the treatment of NTDs, as summarised in Table 2. Many of these AMPs have shown to be promising candidates for the development of therapeutic agents in the battle against NTD infections. However, many obstacles still need to be overcome, including the cytotoxic effects of some peptides and challenges regarding bio-availability. Structural analogues of natural AMPs and hybrid peptide formulations have shown promising results in lowering cytotoxicity and should therefore be further investigated for the possible treatment of NTDs. Additionally, bio-availability issues may be overcome by utilizing nano- or mircoparticle formulations which can also increase specificity and decrease toxicity associated with some AMPs. Apart from the direct application of AMPs, they may be considered for multi-drug treatment regimens as synergistic interactions have been observed when used in combination with conventional antibiotics. Due to this synergism and different mechanisms of action between AMPs and antibiotics, the probability of inducing pathogen resistance to antibiotics can be drastically reduced. AMPs that are currently in clinical trials for the treatment of other conditions should be investigated as potential candidates for the treatment of NTDs. These AMPs might prove to be effective against NTDs; for example, pexiganan, which is undergoing phase 3 clinical trials for diabetic foot ulcers, served as template for the development of an arginine-rich variant that displays potent anti-leishmanial activity.

**Table 2:** Summary of selected AMPs that are associated with or display activity against neglected tropical diseases.

Type	Infection (Causative Agent)	AMP	Source	Notes	Ref
Bacterial	**Leprosy** (*Mycobacterium leprae*)	Human β-defensin 3	*Homo sapiens* (Human)	Up-regulated during a leprosy type 1 infections	[106]
		LL-37	*Homo sapiens* (Human) *Pan troglodytes* (Chimpanzee)	*M. leprae* inhibits *CAMP*—the gene encoding for LL-37	[116]
		Hepcidin	*Homo sapiens* (Human)	Involved in the degradation of ferroportin in multibacillary lesions	[118]
	**Trachoma** (*Chlamydia trachomatis*)	SMAP-29	*Ovis aries* (Sheep)	Reduced inclusion number at concentration of 10 µg/mL. Compromised integrity of extracellular elementary body	[130]
		Melittin	*Apis mellifera* (Honey bee)	Direct cytotoxic effect on *C. trachomatis*. Hindering normal process of intracellular development by lowering the transdermal potential and disrupting the adhesion of the bacteria to the cell	[133]
		Cyto-insectotoxin 1a	*Lachesana tarabaevi* (Central Asian ant spider)	Inhibit *C. trachomatis* infection at an early stage, with higher efficacy than melittin and negligible effect on cell viability	[134]
Parasites	**Chagas disease** (*Trypanosoma cruzi)*	Melittin	*A. mellifera* (Honey bee)	Induced morphological alterations in the different developmental forms of the parasite, which could be characterized as apoptosis and autophagy.	[145]
		Dermasiptin-01	*Phyllomedusa oreades* (Tree frog)	Lytic activity	[149]
Parasites	**Chagas disease** (*Trypanosoma cruzi)*	Tachyplesin I	*Tachypleus tridentatus* (Horseshoe crab)	Able to kill *T. cruzi* epimastigote and promastigotes at micromolar concentrations.	[151]
	**Human African Trypanosomiasis** (*Trypanosoma brucei*)	Temporin-SHd	*Pelophylax saharica* (Sahara frog)	Growth inhibitory activity against *T. brucei* and *T. cruzi*	[155]
		BMAP-27	*Bos Taurus* (Cattle)	Inhibited both life cycles of both *T. brucei* at low micromolar concentrations	[157]
	**Leishmaniasis** (*L. donovani*, *L. major*, *L. tropica*, *L. Mexicana* and *L. panamensis*)	Temporin A and B	*Rana temporaria* (European common frog)	Active against insect and mammalian stages at 15-25µM concentrations. Rapid disruption of plasma membrane potential and decrease in intracellular ATP levels	[160]
		Dermaseptin S4	*Phyllomedusa sauvagii*, (South America Sauvages leaf frog)	Potent lysis for *L. major* promastigotes.	[51]
		Dermasiptin-01	*Phyllomedusa oreades* (tree frog)	Active against *L. infantum* promastigotes by membrane damage and flagella alterations	[164]
		Gomesin	*Acanthoscurria gomesiana* (Tarantula spider)	Decreased the viability of *L. amazonensis* promastigotes with little activity against human erythrocytes	[166]
		Indolicidin	Bovine leukocytes	Membrane effects and able to induce autophagy in *L. donovani*	[167]
Parasites	**Leishmaniasis** (*L. donovani*, *L. major*, *L. tropica*, *L. Mexicana* and *L. panamensis*)	Thionins	*Triticum aestivum* (Wheat)	HNP1–3 can bind to *Onchocerca volvulus* surfaces and is known to mediate the macrophage response against other micro-organisms by the release of IFN-γ and TNF-α	[200]
Helminths	**Onchocerciasis** (**River blindness**) (*Onchocerca volvulus*)	HNP1-3	*Homo sapiens* (Human)	Proved effective against *L. donovani* promastigotes in the low micromolar range, membrane permeability	[168]
		Galectin Peroxidoxin-2	*Onchocerca ochengi* (Black fly)	Excretory/secretory products of *Onchocerca volvulus* with significant antibacterial activity against *E. coli*	[11]
		ALT-1 (all peptides are AMP precursors)	*Onchocerca volvulus* (Roundworm)	The peptides Galectin, Peroxidoxin-2 and ALT-1 are all AMP precursors	
Parasites	**Dengue viral disease** (family *Flaviviridae*)	Latarcin	*Lachesana tarabaevi* (Ant spider)	Able to inhibit the replication of dengue viruses in *in vitro* cultured cells	[232,232]
	**Rabies** (*Lyssavirus*)	C2, C6, C8 and P16	Synthetic	Synthetic AMPs based on naturally according peptides C2, C6, C8 and P16 peptides exhibited strong rabies virus inhibitory properties	[252]

Currently, the greatest obstacle for the use of AMPs, which especially affects its use in the treatment of NTDs, is the cost of the large-scale synthesis of these peptides. Additionally, for large-scale application, isolation from natural sources is also not a viable option. Progress has been made in developing DNA recombinant methods to successfully synthesise and purify AMPs for therapeutic application in a cost effective manner [258,259,260], but the commercial feasibility of these methods still need to be evaluated. Also, because ribosomally synthesised AMPs are expressed by single genes, they may be considered for use in gene therapy for introduction directly into infected tissue. This could significantly reduce the cost associated with the large-scale production and purification of AMPs.

In conclusion, AMPs are effective against a variety of infectious diseases including NTDs. AMPs offer innovative treatment possibilities as they can be used as single anti-microbial agents and in combination with conventional antibiotics, as well as immunomodulating agents. Future research should focus on addressing the issues related to toxicity and challenges associated with mass manufacturing.
