# Correction: Lewies, A., *et al.* The Potential Use of Natural and Structural Analogues of Antimicrobial Peptides in the Fight against Neglected Tropical Diseases. *Molecules* 2015, *20*, 15392–15433

**DOI:** 10.3390/molecules200916757

**Published:** 2015-09-14

**Authors:** Angélique Lewies, Johannes F. Wentzel, Garmi Jacobs, Lissinda H. Du Plessis

**Affiliations:** Centre of Excellence for Pharmaceutical Sciences (PHARMACEN), North-West University, Potchefstroom 2520, South Africa; E-Mails: 20577966@nwu.ac.za (A.L.); 20134045@nwu.ac.za (J.F.W.); 22119027@nwu.ac.za (G.J.)

The authors wish to make the following corrections to this paper [1]:

The author names need to be amended: “Lewies Angélique” should be “Angélique Lewies”; “Wentzel Johannes Frederik” should be “Johannes F. Wentzel”; “Jacobs Garmi” should be “Garmi Jacobs”; “Du Plessis Lissinda Hester” should be “Lissinda H. Du Plessis”.

The correct author names should be: Angélique Lewies, Johannes F. Wentzel, Garmi Jacobs and Lissinda H. Du Plessis.

The authors would like to apologize for any inconvenience caused to the readers by these changes.
